# Anxiolytic, Antidepressant, and Anticholinesterase Effects of Essential Oil from *Myrcia sylvatica* (G.Mey.) DC

**DOI:** 10.3390/biom15010110

**Published:** 2025-01-12

**Authors:** Antônio Quaresma da Silva Júnior, Mariana Maciel Garcia, Wanderson da Silva Farias, Deise Juliane dos Anjos de Sousa, Adenilson de Sousa Barroso, Pablo Luis Baia Figueiredo, Gabriela B. dos Santos, Ricardo Bezerra de Oliveira, Rosa Helena Veras Mourão

**Affiliations:** 1 Programa de Pós-Graduação em Biodiversidade e Biotecnologia da Rede Bionorte, Universidade Federal do Pará, Belém 66075-110, PA, Brazil; antoniojuniort6@hotmail.com (A.Q.d.S.J.); ricardo.oliveira@ufopa.edu.br (R.B.d.O.); 2 Laboratório de Bioprospecção e Biologia Experimental, Universidade Federal do Oeste do Pará, Santarém 68040-255, PA, Brazil; mariana.maciel.garciaa@gmail.com (M.M.G.); wandersonfarias@hotmail.com (W.d.S.F.); juli.dosanjos@hotmail.com (D.J.d.A.d.S.); adenilson.barroso@ufopa.edu.br (A.d.S.B.); 3 Programa de Pós-Graduação em Recursos Naturais da Amazônia, Universidade Federal do Oeste do Pará, Santarém 68040-255, PA, Brazil; 4 Laboratório de Química dos Produtos Naturais, Universidade do Estado do Pará, Belém 66095-015, PA, Brazil; 5 Brazil Programa de Pós-Graduação em Ciências da Saúde, Universidade Federal do Oeste do Pará, Santarém 68040-255, PA, Brazil

**Keywords:** central nervous system, aromatherapy, anxiety, depression, Alzheimer’s disease

## Abstract

Aromatic plants are rich sources of essential oils (EOs), recognized for their therapeutic properties due to their diversity of phytochemicals. This study investigated the anxiolytic and antidepressant effects of *Myrcia sylvatica* essential oil (MsEO) through inhalation in an animal model and its in vitro anticholinesterase (AChE) activity. The EO was obtained by hydrodistillation, and its volatile constituents were analyzed by GC-MS. Swiss mice were exposed to doses of 0.1%, 1%, and 2% of the EO via an inhalation apparatus. The anxiolytic activity was assessed using the elevated plus maze and light–dark box tests, while antidepressant activity was evaluated using the tail suspension and forced swimming tests. To examine potential side effects, the animals were subjected to rotarod, Y-maze, and Morris water maze tests to assess motor coordination, memory, and learning. Anticholinesterase activity was determined by direct bioautography and colorimetry based on the Ellman method. The results demonstrated that inhalation of MsEO at doses of 0.1% and 1% significantly reduced anxiety and depressive-like behaviors without impairing memory, learning, or motor coordination in the animals. Moreover, MsEO inhibited acetylcholinesterase with an IC_50_ of 0.47 μg/mL. These findings suggest that MsEO has potential therapeutic applications for anxiety and depression disorders, with additional anticholinesterase activity warranting further investigation in cognitive-related conditions.

## 1. Introduction

Anxiety and depression are major global public health concerns, affecting approximately 300 million people worldwide. These disorders impair individuals’ quality of life and burden healthcare systems substantially [1]. Conventional treatments often involve psychotropic drugs, such as anxiolytics and antidepressants, which, despite their effectiveness, are frequently associated with adverse side effects [2]. Consequently, there is a growing interest in identifying alternative therapeutic agents derived from natural products.

In addition to being prevalent mental health disorders, anxiety and depression often coexist with neurodegenerative conditions, such as Alzheimer’s disease (AD). Research indicates that up to 80% of individuals with AD experience symptoms of anxiety and depression, which may exacerbate cognitive decline and significantly impact quality of life [3,4]. Furthermore, the interplay between emotional and cognitive disorders is complex and bidirectional: anxiety and depression can precede or accelerate neurodegeneration. At the same time, the progression of conditions like AD may heighten emotional disturbances [5,6]. Therefore, addressing cognitive and emotional symptoms is critical in developing comprehensive therapeutic approaches.

Essential oils (EOs) from aromatic plants have gained attention for their psychoactive properties and potential therapeutic benefits in treating mental health disorders [7]. Specific constituents in EOs, such as terpenes, may exhibit anxiolytic, antidepressant, and anticholinesterase effects, supporting emotional and cognitive well-being [8,9]. Among these the essential oil of *Myrcia sylvatica* (G.Mey.) DC. (Myrtaceae), a native Amazonian plant, has shown promising therapeutic properties, including antioxidant and anti-inflammatory activities [10,11].

*Myrcia sylvatica* is particularly noteworthy due to the unique chemical composition of its essential oil, which lacks a substantial major component but instead comprises a diverse array of volatile compounds, including monoterpenes and sesquiterpenes. Previous studies have highlighted the biological activities of these constituents, including antimicrobial, anti-inflammatory, and neuroprotective effects [12,13]. This chemical complexity makes the oil an intriguing research subject, as its pharmacological effects are likely due to synergistic interactions among its components rather than the dominance of a single compound.

This study aimed to investigate the potential anxiolytic and antidepressant effects of *Myrcia sylvatica* essential oil (MsEO) when administered via inhalation in an animal model. Neurobehavioral tests, such as the elevated plus maze and forced swimming test, were used to assess these effects. Additionally, the in vitro anticholinesterase activity of MsEO was evaluated to explore its potential application in cognitive disorders, particularly those with comorbid symptoms of anxiety and depression. By correlating these findings, we aim to provide a clearer understanding of how MsEO may contribute to addressing the multifaceted nature of neurodegenerative and emotional disorders.

## 2. Materials and Methods

### 2.1. Plant Material and Essential Oil Extraction

Leaves of *Myrcia sylvatica* were collected in the Santa Rosa community (coordinates S 02° 30.464′ W 054° 50.931′), Everaldo Martins Road, Km 21, which connects the city of Santarém to the village of Alter do Chão, Pará, Brazil. A voucher was deposited in the herbarium of EMBRAPA/Eastern Amazon, Belém, Pará, Brazil, under the registration number IAN184696. The leaves were dried at 25 °C, ground, and subsequently used for essential oil extraction.

The MsEO was obtained by hydrodistillation in a modified Clevenger apparatus (Maia and Andrade, 2009) for 120 min. The EO was dehydrated with anhydrous sodium sulfate (Na_2_SO_4_) and stored in amber glass containers under refrigeration until use. The yield of the EO was calculated according to Santos et al. (2004) [14], based on the moisture-free biomass, using the ratio between the volume of oil obtained and the plant biomass used in the extraction.

### 2.2. Essential Oil Analysis

The essential oil was analyzed by a gas chromatography system coupled to a mass spectrometer (GCMS-QP2010 Ultra, Shimadzu Corporation, Tokyo, Japan), equipped with an auto injector (AOC-20i) and CGMSsolution software (Version 4.20), which contains spectra libraries [15] including FFNSC 2 [16]. A fused silica capillary column (Rxi-5ms, Restek Corporation, Bellefonte, PA, USA) of 30 m × 0.25 mm (diameter) × 0.25 µm (film thickness), coated with 5% diphenyl dimethylpolysiloxane was used as stationary phase. The analysis conditions were as follows: helium drag gas (99.995%); split ratio mode at 1:20; injection of 1 µL of the sample (3 µL of the essential oil in 500 µL of hexane); ionization energy by electronic impact (EI) 70 eV; injector temperature: 250 °C; oven temperature program: 60–240 °C; ion source temperature: 200 °C; transfer line temperature: 250 °C.

Quantitative data on the volatile constituents were obtained via peak area normalization using a gas chromatograph (GC 6890 Plus series, Agilent, Santa Clara, CA, USA) coupled to a flame ionization detector (FID), which was operated under similar conditions to the GC-MS system. The mass spectra were obtained by automatic scanning at 0.3 scans/second, with mass fragments of 35–400 *m*/*z*. The compounds found in the ion chromatograms were identified by comparing the mass spectra (molecular mass and fragmentation pattern) with those found in the system’s CGMSsolution library and by comparison with the retention indexes. The linear equation of Van den Dool and Kratz (1963) was used to calculate the volatile components, with the use of a standard homologous series of C8-C20 n-alkanes (Sigma-Aldrich, St. Louis, MO, USA).

### 2.3. Animals

For the animal tests, Swiss male *Mus musculus*, approximately 60 days old and weighing between 35 and 45 g (*n* = 160), were used. The animals were provided by the Laboratory of Bioprospecting and Experimental Biology at the Federal University of Western Pará (UFOPA). They were housed in polypropylene cages, maintained at room temperature (25 ± 2 °C), with a controlled light–dark cycle of 12 h, and had ad libitum access to water and food. The research project was approved by the Animal Ethics Committee (CEUA/UFOPA), under Protocol No. 10004-2017.

### 2.4. Drugs and Treatment

To evaluate the MsEO effects, three animal groups, referred to as EMS, were exposed to doses of 0.1%, 1%, and 2% via inhalation (i.h.) using an inhalation apparatus. To validate the employed pharmacological procedures, the positive control group (PC) was administered the conventional anxiolytic diazepam (Compaz, Brazil—5 mg/kg) or the antidepressant imipramine (Funed, Brazil—15 mg/kg), and the negative control group (NC) was administered mineral water orally (p.o.), 60 min prior to the behavioral tests. These groups were also exposed to the inhalation apparatus, where a hydroalcoholic solution (20%) was administered.

### 2.5. Inhalation Apparatus

The inhalation apparatus consists of an acrylic box (36 × 30 × 29 cm), with the anterior and posterior walls featuring four 2 cm diameter holes. Cotton swabs soaked with the solutions to be evaluated were placed at these locations. The removable lid of the box has a plate with 30 ventilation holes, each 0.4 cm in diameter, to allow airflow. Each cotton swab was loaded with 2 mL of the solution according to the treatment group, and the eight swabs containing the essential oil (OE) were replaced every 10 min to maintain a homogeneous vapor concentration. The animals were placed individually in the apparatus for 5 min [17]. After each exposure, the inhalation apparatus was cleaned with a 10% ethanol solution. Following the treatment, the animals were subjected to their respective behavioral tests.

### 2.6. Behavioral Tests

#### 2.6.1. Evaluation of Anxiolytic Activity

##### Elevated Plus Maze

The apparatus consists of a platform with two open arms (30 × 5 cm) perpendicular to two closed arms (30 × 5 × 25 cm). The maze height from the floor is 45 cm, and the open arms have a border of 0.5 cm [18]. After the treatments, the animals were individually placed at the maze’s center, facing one of the closed arms. They were then observed for 5 min, during which their behavior was recorded on video. The traditional parameters for this model were recorded: the number of entries and the time spent in the open arms. An entry into the arm was considered when the animal placed all four paws inside the arm [19,20]. The apparatus was cleaned with 10% ethanol solution after each exposure.

##### Light-Dark Box Test

The apparatus used was an EP 158 Insight^®^ model, made of acrylic with dimensions of 46 × 27 × 30 cm, consisting of a larger light compartment and a smaller dark compartment, with a passageway between them. Mice were individually placed in the light chamber and allowed to explore the apparatus for 5 min, during which their behavior was filmed. The time spent in each compartment was recorded, with the time spent in the illuminated chamber used as a parameter for anxiety [21]. Between each session, the apparatus was cleaned with 10% ethanol solution.

#### 2.6.2. Evaluation of Antidepressant Activity

##### Tail Suspension Test

The animals were suspended by the tail using adhesive tape (approximately 1 cm from the tail tip) at a height of 50 cm above the bench for 6 min, during which the session was recorded on video. The total immobility time for each animal was recorded in seconds. Mice were considered immobile when they hung passively and remained completely still [22].

##### Forced Swimming

A cylindrical container with a height of 21 cm, a diameter of 20 cm, and a water column of 13 cm at a temperature of 25 ± 2 °C was used. The session lasted 6 min, during which the animal’s behavior was recorded on video. The behavior analyzed was the immobility time, which was assessed during the last 4 min of the recording, as the first 2 min showed little immobility and would have interfered with the evaluation of the actual effect of the treatments [23].

#### 2.6.3. Evaluation of Memory and Learning

##### Y-Maze Test

The apparatus consists of a wooden Y-shaped device with arms measuring 10 cm in width, 34 cm in height, and 43 cm in length, with an angle of 120° between the arms. The animals were placed individually in the maze with an accessible exploration time of 7 min and recorded on video. The sequence of entries into the arms of the maze, labeled as A, B, and C, was recorded during the last 5 min. The data were analyzed to determine the number of entries into the 3 arms without repetition (ABC, CAB, BCA, CBA, or ACB). Each mouse’s alternation percentage was defined as the ratio between the actual number of alternations and the possible number (total number of arm entries minus two) multiplied by 100 [24]. Between tests, the entire maze was cleaned with 10% ethanol.

##### Morris Water Maze Test

A blue polyethylene circular container with a diameter of 80 cm and a height of 30 cm was used, previously filled with water at room temperature (25 ± 2 °C) before the training sessions. The container was divided into four imaginary quadrants, marked by four points: North (N), South (S), East (L), and West (O), which served as starting points for the animals during the test. A 10 cm × 20 cm submerged platform was hidden at a fixed location, one centimeter below the water surface. Non-toxic white paint was added to the water to make the surface opaque and prevent the platform from being visible.

A training session was conducted with the animals to help them learn how to locate the submerged platform. Each animal was placed individually in the water tank, facing the edge of the tank, and allowed to swim freely while using the reference points to search for the platform for 2 min. Animals that did not find the platform within the predetermined time were manually placed under the platform for 10 s to visualize the environment. All animals underwent four training sessions, each corresponding to a different starting point (N, S, L, and O). In the test session, which was conducted 24 h after the training session, the time (in seconds) it took for each animal to find the platform from each starting point was recorded [25].

#### 2.6.4. Assessment of Motor Coordination

##### Rotarod Test

The rotarod (EFF 411 Insight^®^) was used at a speed of 10 revolutions per minute. A training session was conducted to allow the animals to adapt to the apparatus, divided into three series of two minutes each, during which the animals were placed back on the rod as many times as necessary within the stipulated time. After 24 h, the animals were subjected to the test, and each animal was placed again on the rotating rod for three series of two minutes. The time each animal remained on the rod was recorded in seconds, and the result was obtained by calculating the average time across the three series. No additional placement on the rod was made if the animal fell [26].

### 2.7. Determination of the Cholinesterase Inhibition

For the in vitro anticholinesterase assays, the enzyme acetylcholinesterase type VI-S, obtained from *Electrophorus electricus* (lyophilized powder, C3389-2Ku, Sigma-Aldrich, batch: SLBZ8573) was used. The eserine (physostigmine) diluted in methanol was used as a standard anticholinesterase inhibitor (Sigma-Aldrich, batch: BCBC4171V). A standard curve was used to define the concentration used in the tests.

#### 2.7.1. Qualitative Test on Thin Layer Chromatography (TLC)

For the qualitative testing of MsEO and the standard drug, an aluminum chromatoplate for TLC (ALUGRAM^®^ Xtra SIL G, silica gel 60, 0.20 mm, Macherey-Nagel) was used based on the direct bioautography method of Marston et al. (2002) [27]. The enzyme was diluted in tris-HCl buffer, 50 mM, pH 7.8 in ultra-pure water to obtain a concentration of 4 U/mL, with the addition of bovine serum albumin (Sigma-Aldrich) at a ratio of 1:1. Essential oil samples were diluted in methanol at a concentration of 100 µL/mL. Physostigmine was used as the positive control at a concentration of 100 µg/mL. For the negative control, methanol was used. The colorimetric reagents of the test were naphthyl acetate (2.5 mg in methanol) and Fast Blue B salt (2.5 mg in ultra-pure water). Both reagents were prepared and mixed immediately before use.

To perform the test, the aliquots of 10 µL of the oil samples and controls were applied on chromatoplates in duplicate and allowed to stand for 24 h for solvent evaporation. Subsequently, the plate was sprayed with the acetylcholinesterase enzyme solution (4 U/mL) and incubated in a humidity test chamber, without direct contact with moisture, at 37 °C for 20 min. Then, the plate was sprayed with a mixture of naphthyl acetate (2.5 mg) and Fast Blue B salt (2.5 mg) solutions to obtain the results. The formation of a purple coloration occurred gradually, after 1 to 3 min.

#### 2.7.2. Quantitative Assay

The assay for quantifying acetylcholinesterase inhibition of the samples was adapted from Ellman’s method [28], with modifications described by [29]. In summary, three buffers were produced for the quantitative test, which were denominated A, B, and C. These were buffer A = 50 mM Tris/HCl, pH 8, dissolved in ultra-pure water; buffer B = 0.1% bovine serum albumin in buffer A; and buffer C = 0.1 M NaCl and 0.02 M MgCl2.6H2O dissolved in buffer A.

In a total volume of 1 mL, 415 µL of buffer A, 10 µL of the essential oil solution (diluted in methanol, buffer, and Tween 80) at different concentrations (100, 50, 25, 12.5, 6.25 and 3.12 ug/mL), and 75 µL of acetylcholinesterase enzyme, containing 0.2 U/mL, were added. The samples were then incubated for 15 min at 25 °C. After incubation, 75 µL of a solution of 1.83 mM AChI (acetylthiocholine iodide) (Sigma-Aldrich, Steinheim, Germany) and 425 µL of 3 mM DTNB (5,5′-dithiobis[2-nitrobenzoic acid]) (Sigma-Aldrich, Steinheim, Germany) were added and the mixture was incubated for 30 min at 25 °C under a light source. The absorbance of the mixture was measured at 412 nm in a UV spectrophotometer (NOVA, 3300). Physostigmine was used as the standard drug, and a dilution solution was used as negative control (buffer A, methanol, and Tween 80 at a ratio of 2:2:1). The percentage of inhibition of enzyme activity was calculated according to the equation % = [(A0 − A1)/A0] * 100, where A0 was the absorbance of the control without the essential oil and A1 was the absorbance of the essential oil sample at different concentrations. All tests were performed in triplicate. The sample concentration that provided 50% inhibition (IC_50_) was obtained by constructing graphs of the percentages of inhibition versus the concentration of the inhibitor. The non-linear regression parameters for the curve were plotted, and the IC_50_ values were obtained using the Microsoft Excel 2019 software.

### 2.8. Statistical Analysis

The results were expressed as the mean ± standard deviation of the results for the respective experiments, using Analysis of Variance (ANOVA) and Tukey’s test, where *p* < 0.05 was considered statistically significant.

## 3. Results

### 3.1. Volatile Constituents of the Essential Oil of M. sylvatica

The *M. sylvatica* leaf essential oil showed an average yield of 1.12%; its constituents are presented in Table 1. A total of 89.04% of the constituents were identified, with emphasis on the major compounds: β-Selinene (10.06%), *E*-Calamenene (6.38%), *ar*-Curcumene (6.25%), 1,10;7,10-Bisepoxy-1,10-seco-calamenene (4.92%), *ar*-Tumerol (4.54%), and Cadalene (4.24%).

### 3.2. Evaluation of the Anxiolytic Activity of M. sylvatica Essential Oil

#### 3.2.1. Elevated Plus Maze

**Figure 1:**
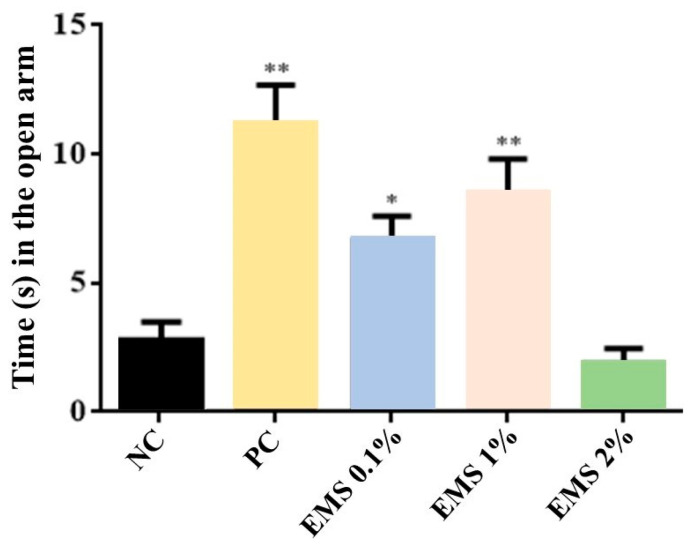
Time (in seconds) spent in the open arms of the elevated plus maze by animals exposed for 5 min to inhalation of *Myrcia sylvatica* essential oil at doses of 0.1%, 1%, and 2% (2 s) (*n* = 8). Values represent the mean ± standard deviation. * *p* < 0.05, ** *p* < 0.01, compared to the negative control group using ANOVA and Tukey’s test. NC = negative control, PC = positive control. EMS: *Myrcia sylvatica* essential oil.

**Figure 2:**
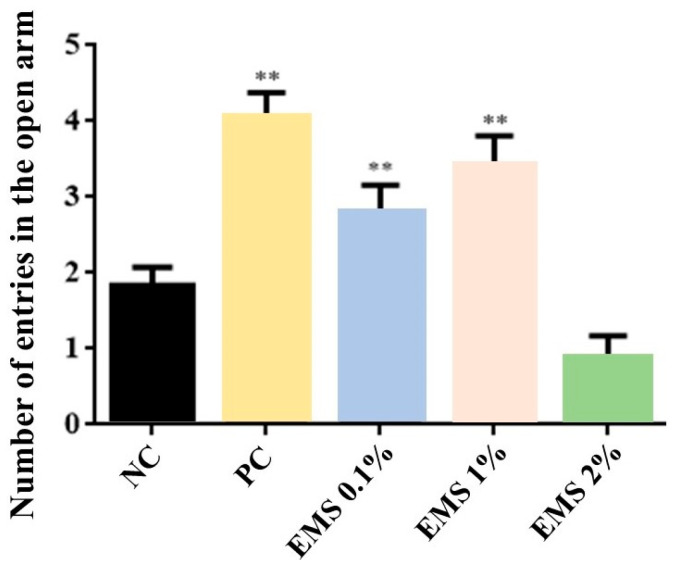
Number of entries into the open arms of the elevated plus maze by animals exposed for 5 min to inhalation of *Myrcia sylvatica* essential oil at doses of 0.1%, 1%, and 2% (*n* = 8). Values represent the mean ± standard deviation. ** *p* < 0.01, compared to the negative control group using ANOVA and Tukey’s test. NC = negative control, PC = positive control. EMS: *Myrcia sylvatica* essential oil.

MsEO treatment significantly increased the time spent in the open arms (at doses of 0.1% [7 s] and 1% [9 s]) [ANOVA: F(4) = 19.77, *p* < 0.05] (Figure 1) and the number of entries into the open arms [ANOVA: F(4) = 19.73, *p* < 0.05] (Figure 2) at doses of 0.1%(3 entries) and 1% (3.5 entries) (*p* < 0.05 vs. negative control [1.8 entries]). However, the 2% dose (1 entry) did not show a significant effect (*p* > 0.05).

Comparing the essential oil-exposed groups to the diazepam-treated group, the standard drug (positive control, PC) in the tests, the 1% dose did not show a significant difference (*p* > 0.05) in both parameters analyzed (Figure 1 and Figure 2).

#### 3.2.2. Light–Dark Box

**Figure 3:**
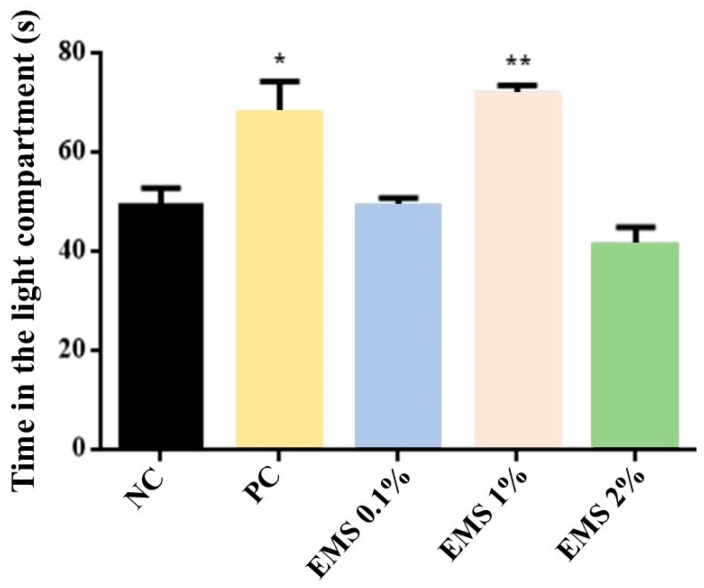
Time spent (seconds) in the light compartment of the light–dark box test by animals exposed to 5 min of inhalation of *Myrcia sylvatica* essential oil at doses of 0.1%, 1%, and 2% (*n* = 6). Values represent the mean ± standard deviation. * *p* < 0.05, ** *p* < 0.01, compared to the negative control group using ANOVA and Tukey’s Test. NC = negative control, PC = positive control. EMS: *Myrcia sylvatica* essential oil.

As shown in Figure 3, the time spent in the light compartment significantly increased [ANOVA: F(4) = 6.75, *p* < 0.05] when exposed to the MsEO at the 1% dose (76 s) (*p* < 0.05 vs. negative control). However, the 0.1% (49 s) and 2% (39 s) doses did not show a significant effect (*p* > 0.05 vs. negative control). When compared to the PC group (71 s), it was observed that the 1% dose did not show a significant difference (*p* > 0.05).

### 3.3. Evaluation of the Antidepressant Activity of Myrcia sylvatica Essential Oil

#### 3.3.1. Tail Suspension Test

**Figure 4:**
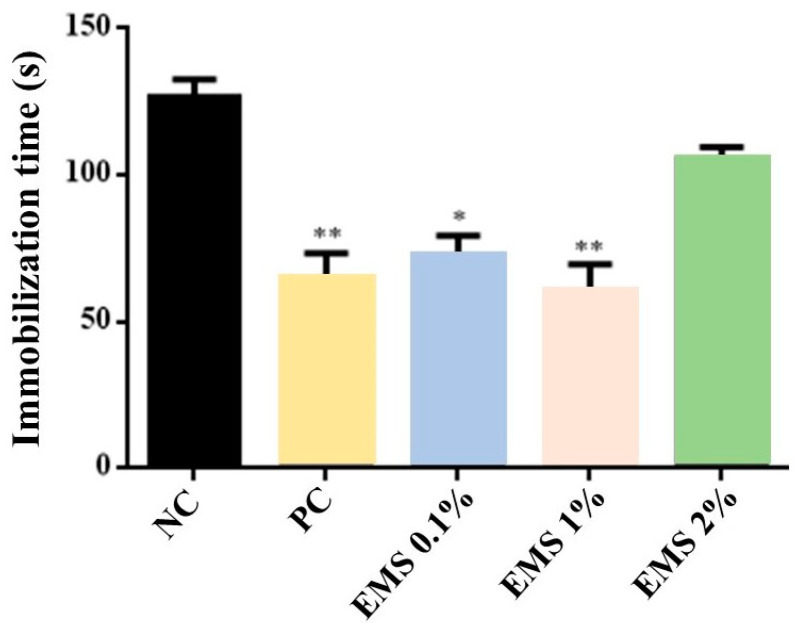
Immobility time (seconds) in the tail suspension test of animals exposed to 5 min of inhalation of *Myrcia sylvatica* essential oil at doses of 0.1%, 1%, and 2% (*n* = 5). Values represent the mean ± standard deviation. * *p* < 0.05 ** *p* < 0.01, compared to the negative control group using ANOVA and Tukey’s Test. NC = negative control, PC = positive control. EMS: *Myrcia sylvatica* essential oil.

MsEO treatment significantly decreased [ANOVA: F(4) = 13.49, *p* < 0.05] the immobility time at the doses of 0.1% (75 s) and 1% (64 s) (*p* < 0.05 vs. negative control). However, the 2% (120 s) dose did not show a significant effect (*p* > 0.05) (Figure 4). When compared to the group treated with Imipramine (positive control), it was observed that the 0.1% and 1% doses did not present a significant difference (*p* > 0.05) in relation to the immobility time of the animals.

#### 3.3.2. Forced Swimming Test

**Figure 5:**
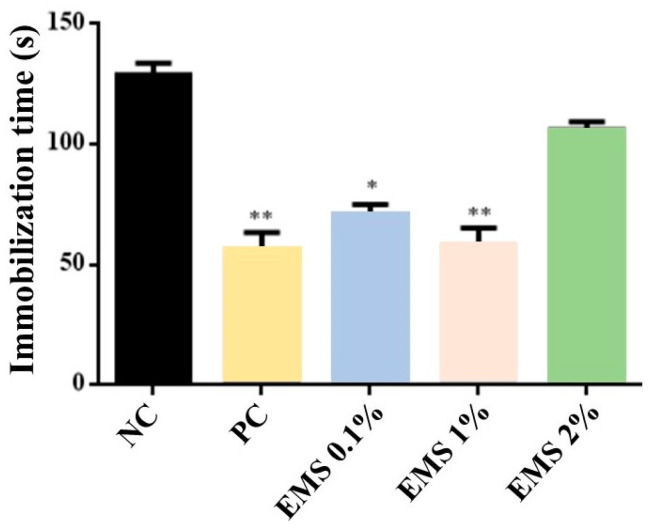
Latency time to immobility during forced swimming of animals exposed to inhalation of *Myrcia sylvatica* essential oil at doses of 0.1%, 1%, and 2% (*n* = 6) for 5 min. The values represent the mean ± standard deviation. * *p* < 0.05, ** *p* < 0.01, compared to the negative control group using ANOVA and Tukey’s test. NC = negative control, PC = positive control. EMS: *Myrcia sylvatica* essential oil.

The treatment with essential oil significantly decreased [ANOVA: F(4) = 9.75, *p* < 0.05] the immobility time at concentrations of 0.1% (65 s) and 1% (53 s) (*p* < 0.05 vs. negative control), with no significant difference (*p* > 0.05) when compared to the CP group. However, the 2% (115 s) concentration showed no significant difference (*p* > 0.05) when compared to the negative control group (Figure 5).

### 3.4. Evaluation of Memory and Learning

#### 3.4.1. Y-Maze

**Figure 6:**
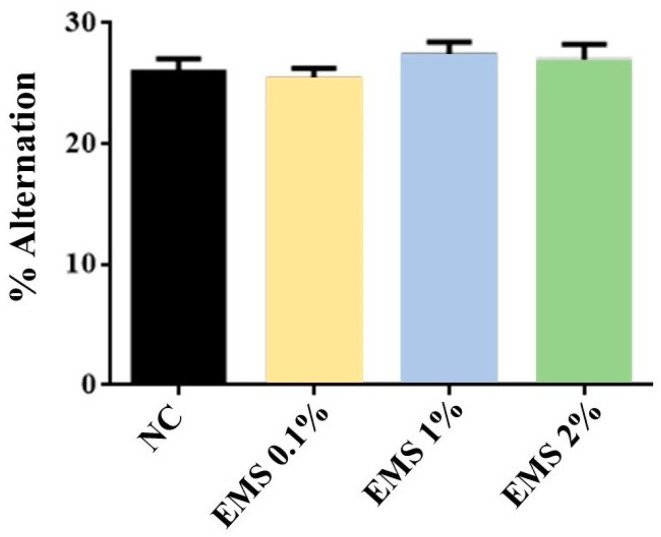
Percentage of alternation in the arms of the Y-maze in animals exposed to 5 min of inhalation of *Myrcia sylvatica* essential oil at doses of 0.1%, 1%, and 2% (*n* = 8). Values represent the mean ± standard deviation. *p* < 0.05; *p* < 0.01, compared to the negative control group using ANOVA and Tukey’s Test. NC = negative control. EMS: *Myrcia sylvatica* essential oil.

MsEO treatment did not show a significant difference [ANOVA: F(3,28) = 0.689, *p* = 0.565] in the percentage of correct sequences when compared to the negative control group (Figure 6).

#### 3.4.2. Morris Water Maze

**Figure 7:**
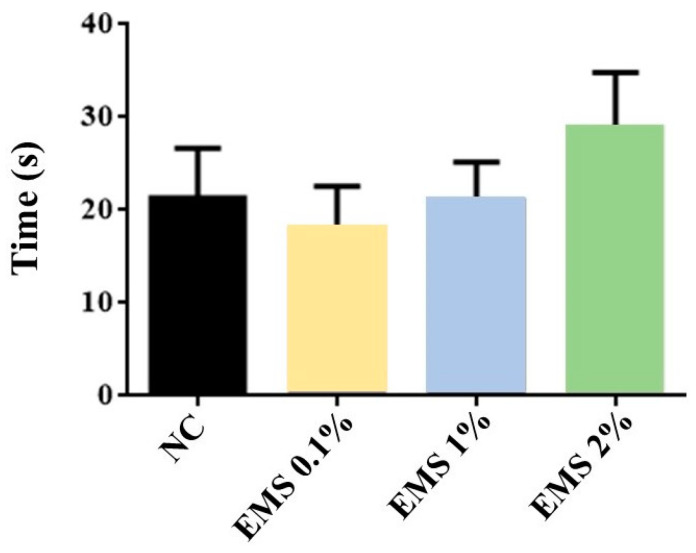
Average time in seconds spent to locate the platform from the four reference points in the Morris water maze of animals exposed to 5 min of inhalation of the essential oil of *Myrcia sylvatica* at doses of 0.1%, 1%, and 2% (*n* = 8). Values represent the mean ± standard deviation. *p* < 0.05, *p* < 0.01, compared to the negative control group using ANOVA and Tukey’s Test. NC = negative control. EMS: *Myrcia sylvatica* essential oil.

As shown in Figure 7, treatment with MsEO did not result in a significant difference [ANOVA: F (3,28) = 0.827, *p* = 0.490] in the average time to locate the platform across the four quadrants, when compared to the negative control group.

### 3.5. Assessment of Motor Coordination

#### Rotarod Test

**Figure 8:**
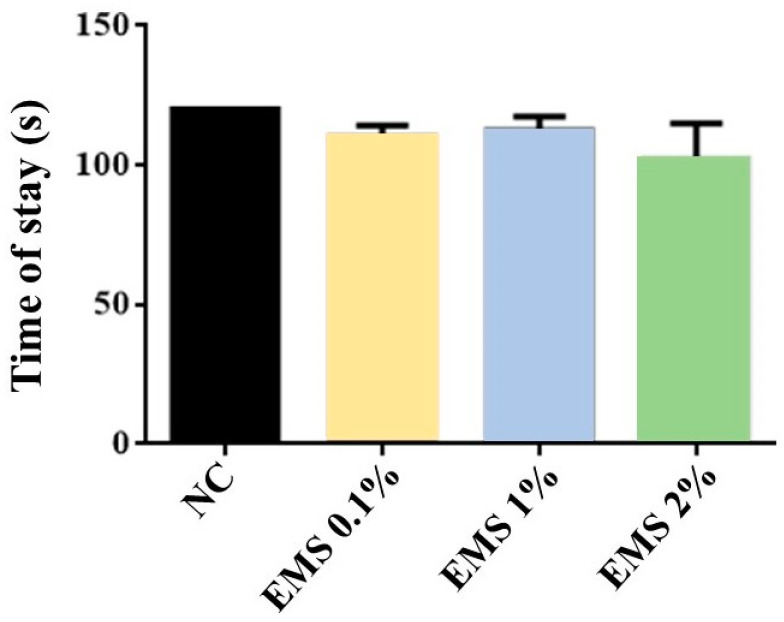
Mean time in seconds spent on the rotarod by animals exposed to 5 min of inhalation of *Myrcia sylvatica* essential oil at doses of 0.1, 1, and 2% (*n* = 8). Values represent the mean ± standard deviation. *p* < 0.05, *p* < 0.01, compared to the negative control group using ANOVA and Tukey’s test. NC = negative control. EMS: *Myrcia sylvatica* essential oil.

MsEO Inhalation did not alter the time spent on the rotarod [ANOVA: F (3,28) = 0.5490, *p* = 0.6529] when compared to the negative control group (Figure 8).

### 3.6. Anticholinesterase Test—Qualitative and Quantitative

**Figure 9:**
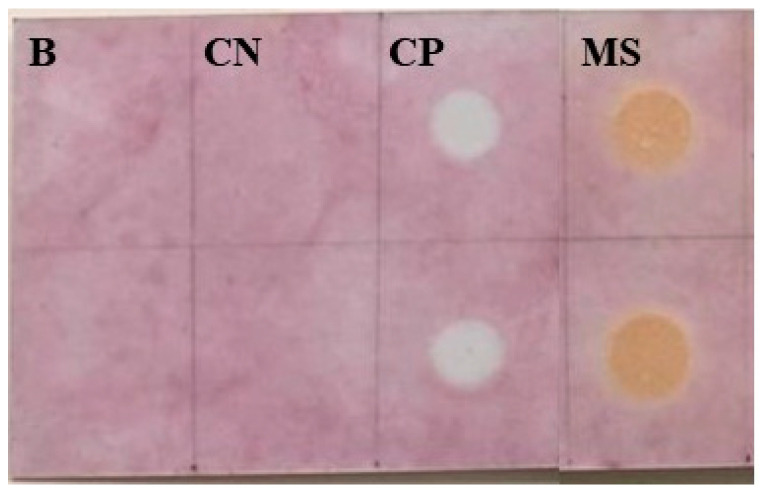
Direct bioautography on silica gel chromatoplates (CCD) of the anticholinesterase activity of the essential oil of *Myrcia sylvatica* and the standard physostigmine. (B) blank, (CN) negative control: methanol, (CP) positive control: physostigmine (100 μg/mL). MS = EO of *M. sylvatica*.

In Figure 9, the inhibition halos of the enzyme acetylcholinesterase are shown from the direct bioautography assay of MsEO, where we can observe the formation of an inhibition halo on the chromatographic plate. Regarding the quantitative assay, the IC_50_ found was 0.47 ± 0.015 μg/mL.

These results indicate that MsEO exhibits strong inhibition against the AChE enzyme. It is worth noting that the anticholinesterase potential of this species has not been described in the literature, making this the first report of the species being identified as an AChE inhibitor.

## 4. Discussion

Central nervous system (CNS) disorders affect millions worldwide, impairing various aspects of human behavior such as thought, emotions, memory, sensations, language, and movement [30]. Anxiety and depression disorders are the most common, affecting around 300 million people globally [31]. Moreover, there is a significant relationship between these disorders and Alzheimer’s disease (AD). Studies show that patients with AD frequently experience symptoms of depression and anxiety, which may worsen disease progression and impact life quality [32,33]. Conventional drugs available for the treatment of these disorders have significant side effects, making adherence difficult for patients, and are considered inadequate [34].

In searching for new therapeutic tools, aromatic plants and their active compounds play an increasingly important role in medicine [35]. Essential oils (EOs) have been the subject of research in different behavioral models and administration methods, proving to be an alternative or complement to conventional anxiolytics and antidepressants [36,37]. These substances may offer a promising approach for managing depressive and anxious symptoms in AD patients, potentially improving their life quality and delaying the progression of symptoms. In this context, the present study evaluated the anxiolytic, antidepressant, and anticholinesterase potential of MsEO through inhalation treatment, simulating aromatherapy, and using animal models in behavioral tests.

In the elevated plus maze test, the MsEO at doses of 0.1% and 1% increased the time spent and the number of entries into the open arms, indicating an anxiolytic effect. This model is widely used to investigate treatments with potential anxiolytic effects in rodents [20,38] and is based on the natural behavior of rodents’ aversion to open and elevated spaces [39,40]. The anxiolytic effect of a drug is related to increased time spent (in seconds) and the number of entries into the open arms [20,40,41].

Complementing this finding, the light–dark box test was used based on the mice’s aversion to brightly lit spaces versus their spontaneous exploratory behavior [42,43]. The anxiolytic effect is related to increased time spent in the light compartment [44,45]. The results showed that MsEO at a dose of 1% increased the time spent in the light compartment. Faturi et al. (2021) [46] found similar results regarding the dose response of the EO of *Citrus sinensis*, where inhalation of the intermediate dose increased the time spent in the light compartment. Thus, the results obtained in both methods indicate that MsEO exhibits an anxiolytic-type response, particularly at the 1% dose.

For pharmacological validation of the elevated plus maze and light–dark box tests, diazepam (5 mg/kg), a conventional anxiolytic, was administered orally, demonstrating its typical effects in both tests. This drug belongs to the class of benzodiazepines, which are CNS depressants. Its pharmacological action potentiates the effect of the inhibitory neurotransmitter gamma-aminobutyric acid (GABA), binding to the GABA-A receptor and increasing the frequency of opening the chloride channel, resulting in an increased influx of chloride through the receptor channel, facilitating rapid inhibitory transmission mediated by GABA and generating sedative and anxiolytic effects [40,47,48].

To evaluate antidepressant activity, the tail suspension and forced swimming tests were used. Although these tests do not fully reflect the symptoms of depression in humans, they are widely used to investigate compounds with antidepressant activity [49]. These tests are based on learned helplessness, where the animal, after an initial period of struggling, becomes immobile, reflecting a state of despair [50]. The administration of antidepressants increases the time the animal attempts to escape, reducing immobility [23,36]. The doses of 0.1% and 1% of MsEO reduced the immobility time, suggesting an antidepressant-like response to inescapable stress. These findings are particularly relevant in Alzheimer’s disease, where patients often exhibit depressive symptoms that can worsen the clinical condition and reduce quality of life [5].

Imipramine (15 mg/kg) was used for pharmacological validation of the tests, showing the expected effect of reducing immobility rates. As a tricyclic antidepressant, imipramine preferentially blocks serotonin and norepinephrine transporters, increasing the levels of these neurotransmitters in synapses [51].

Deficits in motor coordination and memory may accompany the anxiolytic and antidepressant effects of conventional medications [36,43]. Therefore, it was important to investigate whether the inhalation of MsEO affected motor coordination, memory, and learning, using the Y-maze, Morris water maze, and rotarod tests.

In the Y-maze test, inhalation of MsEO did not affect short-term memory based on exploring a new environment [52]. Similarly, in the Morris water maze, the EO did not interfere with spatial memory or learning, indicating that the rodents could learn how to find the platform to escape from the water [53]. These results are particularly relevant in the context of AD, which is characterized by cognitive and memory deficits, especially in the early stages. The preservation of short-term memory and spatial memory is crucial, as these cognitive domains are often affected in patients with Alzheimer’s. The fact that MsEO did not show any negative interference in memory tests suggests a therapeutic potential that could be explored to develop interventions that improve cognitive function or delay the progression of symptoms in individuals with Alzheimer’s. Therefore, using natural substances like EO could represent a promising alternative to managing cognitive deficits associated with the disease.

The rotarod test evaluates the integrity of motor function, assessing potential adverse effects on motor coordination [54]. In this test, no loss in motor performance was observed in the animals at any of the three doses of EO administered. Although the 2% dose did not show any anxiolytic or antidepressant effect, it did not interfere with motor coordination or the processes of memory and learning. These results are encouraging, as they suggest that MsEO could be a promising alternative with antidepressant effects without compromising essential cognitive functions, which is especially relevant in the context of neurodegenerative diseases such as Alzheimer’s.

The MsEO administered via inhalation at 0.1% and 1% demonstrated anxiolytic and antidepressant potential without negatively affecting memory, learning, or motor coordination processes. In this administration method, the volatile substances reach the olfactory receptors, which are directly connected to the central nervous system, sending messages to the limbic system, where anxiety and emotions are often generated [55], leading to the release of neurotransmitters such as serotonin, endorphins, and norepinephrine [56], which communicate with various systems in the body, affecting heart rate, blood pressure, and hormone release [57].

Volatile substances can also be absorbed by the nasal mucosa or through pulmonary blood vessels, spreading throughout the body and potentially crossing the blood–brain barrier, modulating signaling pathways involved in anxiety and depression [36,58]. This modulation is especially relevant in the context of Alzheimer’s disease, where emotional dysfunction and anxiety are often observed in patients, exacerbating cognitive deficits.

The fact that MsEO does not affect memory suggests a therapeutic potential that could be explored to improve the quality of life of individuals with Alzheimer’s, offering a natural alternative to conventional interventions. Several studies have demonstrated the anxiolytic and antidepressant potential of essential oils, such as Cananga odorata [59] and *Lavandula angustifolia* [60]. The effectiveness of essential oils is related to their distinct chemical composition [61], making chemical characterization crucial to ensure the standardization and reproducibility of their pharmacological effects [40].

The results of this study demonstrate the neuropharmacological potential of *Myrcia sylvatica* essential oil, though the absence of a clear dose–response relationship warrants further investigation. This characteristic may stem from the complex composition of the oil, where synergistic or antagonistic interactions between its constituents influence the observed effects. While statistically significant, the biological relevance of these findings must be interpreted cautiously, particularly given the multifactorial nature of the tested models. Future studies focusing on isolated components, their interactions, and validation in alternative models are necessary to understand better the mechanisms underlying these effects and confirm their clinical applicability.

MsEO demonstrated significant anticholinesterase activity, with an IC_50_ value of 0.47 µg/mL. This activity is particularly relevant in the context of neurodegenerative diseases such as Alzheimer’s disease, where the inhibition of acetylcholinesterase (AChE) is a common therapeutic strategy. AChE is responsible for the degradation of the neurotransmitter acetylcholine, which is crucial for cognitive functions such as memory and learning [62]. The ability of MsEO to inhibit AChE suggests a promising potential for modulating acetylcholine levels, which could contribute to improving cognitive symptoms associated with Alzheimer’s disease [63]. Furthermore, the effectiveness of essential oil, when compared to synthetic inhibitors, could offer a safer alternative with fewer side effects since many conventional drugs are associated with complications such as toxicity and drug interactions [6]. Investigating the chemical composition of the EO could shed light on the compounds responsible for this anticholinesterase activity, allowing for standardization and the use of the essential oil in therapeutic strategies for treating cognitive disorders.

The EO extracted from *M. sylvatica* leaves has a yield of approximately 1%, which is considered favorable for industrial-scale extraction [64]. It has a moss-green color, with spicy and woody fragrance notes [65], and a complex chemical composition, characterized by the presence of volatile compounds in low concentrations, with the major constituents being β-selinene (10.06%), (*E*)-calamene (6.38%), and *ar*-curcumene (6.25%). Although these compounds are present in low concentrations, it is possible that their synergistic effect contributes to the pharmacological activity of the essential oil. Future studies should investigate the role of individual components and their interactions in producing the observed effects.

The synergy between components of essential oils is a well-documented phenomenon in the literature, where the combination of bioactive substances can potentiate therapeutic effects compared to the effects of each isolated component [61,66]. Previous studies have indicated that the therapeutic action of essential oils, such as *Cananga odorata* and *Lavandula angustifolia*, can be significantly modulated by the interaction between their constituents, rather than depending on a single dominant compound [67,68]. This synergistic interaction may explain the efficacy of MsEO in anxiety, depression, and anticholinesterase models, despite the absence of a clear major component. Therefore, investigating the interaction between the components of MsEO and their impact on pharmacological effects is essential to understand better its therapeutic potential, especially in neurodegenerative diseases like Alzheimer’s. Thus, no studies have been found in the literature on the anxiolytic and antidepressant effects of these isolated compounds or the anticholinesterase activity of this essential oil.

Several factors such as seasonality, soil and climate conditions, and plant phenology can affect the yield and chemical composition of essential oils, which in turn may influence their biological activity [69,70]. A study on the chemical stability of MsEO during seasonal and circadian periods reported a small quantitative variation between the rainy and dry seasons and at different collection periods. The chemical stability, highly pleasant aroma, significant pharmacological effects (anxiolytic and antidepressant response), and ability to inhibit acetylcholinesterase make this EO a promising therapeutic tool in aromatherapy, both as an adjunct in the treatment of anxiety and depression and in Alzheimer’s disease [71].

Nevertheless, the results should be validated in other systems to confirm the therapeutic potential of MsEO. Limitations such as the small sample size and the absence of a clear dose–response relationship need to be addressed in future studies.

## 5. Conclusions

Our study demonstrated that *M. sylvatica* essential oil exhibits anxiolytic and antidepressant effects in an animal model without compromising the animals’ motor coordination, memory, and learning abilities. This characteristic is particularly relevant, as many conventional drugs used to treat anxiety and depression disorders can cause unwanted side effects, such as cognitive and motor impairment.

The anticholinesterase activity of the oil further strengthens its potential in clinical contexts, especially in the management of neurodegenerative conditions such as Alzheimer’s disease, where modulating acetylcholine levels is crucial for maintaining cognitive functions. Therefore, the inhalation of *M. sylvatica* EO may offer a helpful alternative or complementary option in the prevention or treatment of emotional and cognitive disorders.

## Figures and Tables

**Table 1:** Volatile constituents of *M. sylvatica* essential oil.

Constituents	I.R._Calc_	I.R._Lit_	Conc. (%)
α-Pinene	934	932	0.12
β-Pinene	978	974	0.03
Limona ketone	1130	1131	0.09
Nopinone	1137	1135	0.12
(*E*)-Pinocarveol	1138	1135	0.88
Pinocarvone	1162	1160	0.16
*p*-Mentha-1,5-dien-8-ol	1166	1166	0.73
*p*-Methyl-acetophenone	1183	1179	0.3
α-Terpineol	1191	1186	0.15
Myrtenol	1196	1194	1.12
Cumenol	1203	1996	0.06
Verbenone	1209	1208	0.53
α-Copaene	1377	1374	0.2
(*E*)-α-Bergamotene	1437	1432	0.14
(*Z*)-Muurola-3,5-diene	1451	1448	0.12
β-Santalene	1461	1457	0.11
Dehydrosesquicineole	1471	1469	0.08
(*E*)-Cadina-1(6),4-diene	1475	1475	0.34
*ar*-Curcumene	1484	1479	6.25
β-Selinene	1488	1489	10.06
(*E*)-Muurola-4(14),5-diene	1493	1493	0.28
β-Bisabolene	1509	1505	0.59
(*E*)-Calamenene	1525	1521	6.38
Zonarene	1527	1528	0.42
(*E*)-iso-ɣ-Bisabolene	1529	1528	0.13
(*E*)-Cadina-1,4-diene	1534	1533	0.17
Occidentalol	1542	1543	4.98
α-Calacorene	1544	1544	3.35
β-Calacorene	1564	1564	0.93
*ar*-Tumerol	1588	1588	4.54
Caryophyllene oxide	1584	1582	3.20
1,10;7,10-Bisepoxy-1,10-seco-calamenene	1587	1587	4.92
Guaiol	1603	1600	2.64
Humulene epoxide	1611	1613	2.38
α-Colocalene	1623	1622	0.29
1-*epi*-Cubenol	1630	1627	4.02
Caryophylla-4(12),8(13)-dien-5-β-ol	1637	1639	2.62
Cubenol	1644	1645	1.49
*epi*-α-Muurolol	1647	1640	1.07
Agarospirol	1650	1646	1.44
Isobornyl 5-hydroxy-isobutanoate	1654	1658	1.70
α-Bisabolol oxide	1657	1656	3.04
α-Bisabolol oxide B	1660	1655	4.36
(*Z*)-Calamenen-10-ol	1664	1660	0.55
14-Hydroxy-9-*epi*-(Z)-caryophyllene	1673	1668	0.46
Cadalene	1676	1675	4.24
Mustakone	1679	1679	3.02
α-Bisabolol	1687	1685	3.40
10-*nor*-Calamenen-10-one	1703	1702	0.28
*ar*-Curcumen-15-al	1713	1712	0.33
14-Hydroxy-α-humulene	1719	1713	0.23
Total (%)			89.04

RI_calc_ = Calculated retention time; RI_Lit_ = Retention time from the literature. Conc. (%) = percentage concentration of the constituents.

## Data Availability

The data associated with this study have not been deposited in a publicly available repository; however, they can be provided upon request.
